# A modified McCabe score for stratification of patients after intensive care unit discharge: the Sabadell score

**DOI:** 10.1186/cc5136

**Published:** 2006-12-27

**Authors:** Rafael Fernandez, Francisco Baigorri, Gema Navarro, Antonio Artigas

**Affiliations:** 1 Critical Care Centre, Hospital de Sabadell, Parc Taulí s/n. 08208, Sabadell, Spain; 2 Department of Epidemiology, Hospital de Sabadell, Sabadell, Spain

## Abstract

**Introduction:**

Mortality in the ward after an intensive care unit (ICU) stay is considered a quality parameter, and is described as a source of avoidable mortality. Additionally, the attending intensivist frequently anticipates fatal outcome after ICU discharge. Our objective was to test the ability of a new score to stratify patients according to ward mortality after ICU discharge.

**Methods:**

A prospective cohort study was performed in the general ICU of a university-affiliated hospital. In 2003 and 2004 we prospectively recorded the attending intensivist's subjective prognosis at ICU discharge about the hospital outcome for each patient admitted to the ICU (the Sabadell score), which was later compared with the real hospital outcome.

**Results:**

We studied 1,521 patients with a mean age of 60.2 ± 17.8 years. The median (25–75% percentile) ICU stay was five (three to nine) days. The ICU mortality was 23.8%, with 1,156 patients being discharged to the ward. Post-ICU ward mortality was 9.6%, mainly observed in patients with a Sabadell score of 3 (81.3%) or a score of 2 (41.1%), whereas lower mortality was observed in patients scoring 1 (17.2%) and scoring 0 (1.7%). Multivariate analysis selected age and the Sabadell score as the only variables associated with ward mortality, with an area under the receiver operating curve of 0.88 (95% CI 0.84–0.93) for the Sabadell score.

**Conclusion:**

The Sabadell score at ICU discharge works effectively to stratify patients according to hospital outcome.

## Introduction

Mortality in the ward after intensive care unit (ICU) discharge is considered a quality parameter, and is commonly defined as a source of unexpected or avoidable mortality. Mortality has been reported to range from 6% to 27% [1] and can be related to factors occurring before or after the ICU stay. A worse outcome is associated with the physiological reserve before ICU admission [2], the type of illness, the intensity of care required, and the clinical stability and/or the grade of nursing dependence at discharge [3,4]. These data suggest that keeping at-risk patients in the ICU for a further 48 hours might reduce mortality after ICU discharge by 39% [5]. Accordingly, step-down units may reduce post-ICU mortality by avoiding inappropriate early discharges from the ICU [6]. It is also yet to be determined whether outreach teams have a favourable impact on the ward mortality rate in this specific population [7].

Nevertheless, fatal outcome in the ward after ICU discharge is frequently an anticipated event [8]. A significant number of patients survive the critical illness with sequelae that severely limit the quality of life and with expectations for a full functional recovery. The only tools presently available to predict hospital mortality are the standard severity scores at ICU admission [9], and calibration of these scores after ICU discharge is poor. Our hypothesis was that ward mortality can be more accurately anticipated by the attending intensivists at ICU discharge, as suggested in our preliminary report [10]. The objective of the present study was to analyse post-ICU mortality and the predictive power of a new subjective score at ICU discharge to stratify patients and their hospital outcome.

## Materials and methods

Our Critical Care Department comprises a closed 16-bed medical-surgical ICU and a closed 10-bed step-down unit. The 10 ICU physicians attend in working hours, and are also on duty at night and at weekends, taking care of a balanced amount of patients throughout the year.

In 2002, we added a new predictive score to our standard Critical Care Discharge Form, based on a modification of the McCabe and Jackson score [11]. We transformed the original three-group classification into a four-group model by splitting the 'ultimately fatal' prognosis into a 'long-term' prognosis and a 'short-term' prognosis. This predictive score reflects a subjective prognosis for each patient at discharge, based on the subjective perception of the attending intensivist. The score includes only four options: good prognosis (0 points), poor long-term prognosis (> 6 months) with unlimited ICU readmission (1 point), poor short-term prognosis (< 6 months) with debatable ICU readmission (2 points), and death expected during hospitalisation with ICU readmission not recommended (3 points). The ICU intensivist and ICU resident responsible for a given patient complete this prediction score at discharge by consensus, based on their unique subjective perception during the whole ICU stay. These physicians do not take into account any of the mortality prediction scores commonly used in the ICU (that is, the Acute Physiologic and Chronic Health Evaluation (APACHE) II score and the Mortality Prediction Model score). Their opinion was also influenced in the daily rounds with the whole ICU team. Specific training was minimal, consisting of only one explanatory session prior to the study, but the research investigators were always reachable for specific questions while the study was underway. In case of ICU readmission, only the score at first ICU discharge was taken into account.

A feasibility trial was performed in November and December 2002, and the study included all patients admitted between 2003 and 2004. As the study was an analysis of the Critical Care Center database, informed consent was waived.

The ward team was unaware of the ICU subjective prediction. While communication between the ICU and ward teams as part of the daily routine remained allowed, there was no formal outreach team. The post-ICU outcome was independently recorded. End-of-life issues remained at the discretion of the primary physicians according to the specific Hospital Protocol for Advanced Directives.

The statistical approach was descriptive, using the mean ± standard deviation or percentages and the odds ratio when appropriate. Variables were compared by analysis of variance with Scheffe post-hoc analysis when appropriate, with significance at *P *< 0.05. Categorical variables were analysed by exact Fisher tests. Univariate analysis of hospital mortality was performed with the Kaplan–Meier estimate-of-survival curve. Multivariate analysis of ward mortality was performed by binary logistic regression. The predictive power of the Sabadell score for ward mortality was tested by receiver operating curves, and its calibration was assessed by the Hosmer–Lemeshow statistic.

## Results

There was a total of 1,521 admissions in the ICU in the studied years, with an occupancy ratio of 91%. Almost one-third (408 out of 1521) of ICU patients were transferred to the step-down unit before ward discharge. The mean age of patients was 60.2 ± 17.8 years, and the admission diagnosis was postsurgical in 18%, was cardiac diseases in 30%, and was medical disorders in 52%. The hospital mortality predicted by the APACHE II score was 25.9 ± 24.4%, whereas the ICU mortality was 23.8%. No deaths occurred in the step-down unit so a total of 1,159 patients were transferred to the ward, where 111 (9.6%) finally died and 1,048 (90.4%) were discharged from hospital. Clinical characteristics of the patients in the four prognosis categories at ICU discharge are presented in Table 1, with significant differences between groups with progressively worse values at each associated level of prognosis. The ICU readmission rate did not reach statistical significance, probably because of the few cases in each group, and there were no deaths in the ICU in this small population.

**Figure 1:**
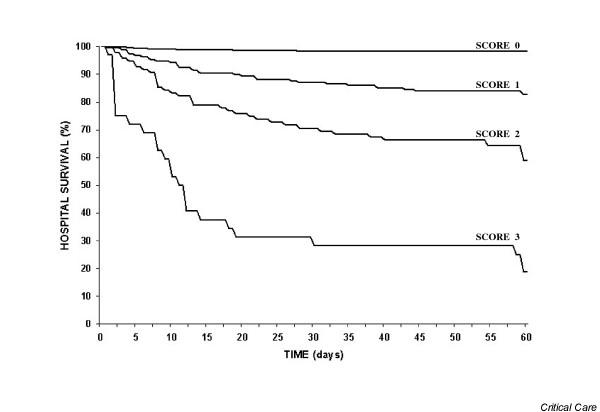
Survival analysis in the ward according to each subjective prognosis. Cox-proportional hazard test showed statistical differences at each score value.

The survival analysis on the ward for each group of subjective prognosis is shown in Figure 1. The ward mortality was 1.7% (95% CI 1.0–2.8) for good prognosis, 17.2% (95% CI 12.5–23.3) for long-term poor prognosis, 41.1% (95% CI 31.7–51.1) for short-term poor prognosis, and 81.3% (95% CI 64.7–91.1) for those patients with expected hospital death (*P *< 0.01). The lack of overlap highlights the good performance of the Sabadell score. A subgroup analysis comparing early (< 7 days) and late (> 7 days) ward mortality showed that 45% of all deaths in the ward occurred in the first week, with no differences among groups.

Table 2 depicts the variables associated with ward mortality according to univariate analysis, whereas the nonsignificant variables were cancer, emergency surgery, acute renal failure, and ICU admission in the previous six month period. Using multivariate analysis, the ward mortality was associated with three significant variables: age, tracheostomy, and APACHE II risk of mortality (Table 3). When we included the categorical Sabadell score in the multivariate analysis, only age and the new score remained independently associated with ward mortality. Each odds ratio for the Sabadell score related to 'good prognosis' as reference value.

**Figure 2:**
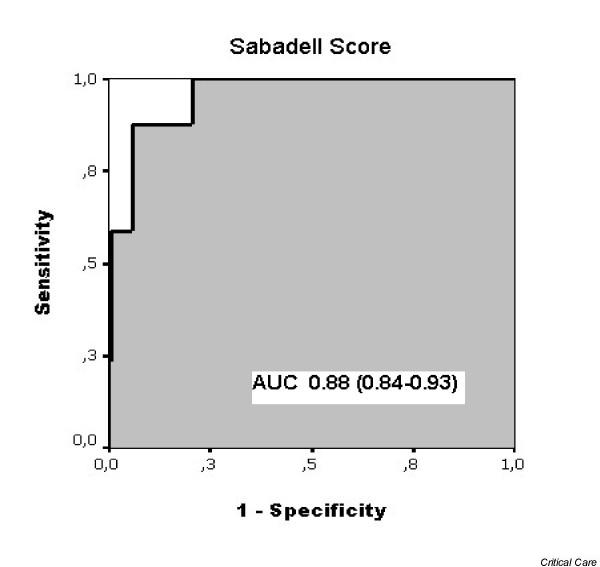
Receiver operating curve for the Sabadell score as a predictive tool for ward mortality after intensive care unit discharge. AUC, area under the curve.

The area under the receiver operating curve for prediction of ward mortality for the Sabadell score was 0.88 (95% CI 0.84–0.93) (Figure 2), with the Hosmer–Lemeshow goodness-of-fit statistics (χ^2 ^= 6.6, significance = 0.58) showing good calibration and discrimination of the model.

## Discussion

Our results suggest that mortality in the ward after ICU discharge mainly affects patients with very poor prognosis according to the subjective perception of ICU physicians. Quality improvement in this area may therefore be restricted to the population with good prognosis or with bad prognosis only in the long term who had a 2–17% likelihood of ward mortality. On the contrary, patients with predicted bad prognosis in the short term, despite a ward mortality > 40%, may be best surveyed by a palliative care team.

The most common approach to date for prognosis of patients after discharge from the ICU is the use of severity scores calculated on admission. Some of these scores, such as the Mortality Prediction Model, take into account the physiological alterations on admission, whereas other scores, such as the APACHE II score, use the worst values within the first 24 hours of ICU admission. Some investigators have tried to improve the ability of these scores, either by customisation according to the case mix or by applying the scores in a sequential mode over the first week of the ICU stay [9]. Nevertheless, due to the need for simplicity, most ICUs still use the original APACHE II severity score as their routine risk-assessment tool. Our data demonstrate that the APACHE II score remains an independent factor associated with ward mortality with a low but significant predictive power. Nevertheless, the inclusion of the new Sabadell score eliminates APACHE II from the previous model described by multivariate analysis. In this new multivariate analysis, age remained the only independent factor that worked with the Sabadell score to construct the model.

Because of the holistic and subjective dimension of the new score, as in the original McCabe score, intensivists probably integrate the previous quality of life and comorbidities with the severity of organ dysfunctions remaining at ICU discharge as a consequence of the acute illness. Another interesting issue is to what degree physicians integrate some implicit knowledge about the health system (that is, the likelihood of survival) with such derangements in a given clinical scenario. Our system, with a step-down unit into the Critical Care Department and with attending intensivists in place, is a very specific feature. As suggested by Daly and colleagues [5], our moderately low post-ICU mortality could be partly explained by the fact that as many as one-third of our ICU patients spend an additional period in our step-down unit facility.

### Limitations of the study

The inclusion period of two years is longer than a standard prevalence observation, but is not sufficiently long to elucidate any trend in outcomes. The single-centre design of this study precludes the direct extrapolation of these results to the wide spectrum of ICUs. The lack of strict definition criteria for expected outcome may reduce the ability to transfer this observation to other ICUs, but, actually, the good results obtained over the two years, despite frequent resident turnover, suggest that the subjective prognosis based on medical common knowledge is reliable. The concordance between data reported over the first year of the study [10] and the global results appear to reinforce the reliability of our analysis. Nevertheless, we as yet have no data about the score in terms of repeatability or agreement between physicians concerning a given patient; these data can be difficult to obtain because the score is based on the unique patient-doctor relationship. The sample size precludes the possibility of finding a difference between physicians in their scoring ability. Moreover, our study did not include the perception of nurses, an issue that has been shown to differ greatly from that of physicians [8,12].

Since all patients who died after ICU discharge died on the ward without ICU readmission and since we do not have access to data to assess whether these deaths occurred with a specific decision to limit ICU readmission or other life-sustaining treatment, it is possible that the Sabadell score subjective prognosis is a self-fulfilling prophecy reflecting a decision of the ICU team about readmission to the ICU. The fact that ward physicians and the majority of the ICU team were unaware of the scoring reduces this possibility, although it may not affect the ability of the attending intensivist to influence care. Given the number of ICU attending physicians, it is unlikely that the ICU attending physician making the subjective prognosis would be responsible for a decision about ICU readmission. As with all prognostic tools, and specifically for subjective ones, the external validity of this assessment should be assessed both in new populations of patients and in the hands of new physicians before it can be generally recommended.

An additional source of bias could be the case mix of our ICU. Our centre is a university-affiliated hospital covering an area of about 420,000 inhabitants in the metropolitan area of Barcelona. The hospital provides emergency care and medical and surgical services except for treatment of burns, cardiovascular surgery, and transplantation. In a recent Spanish multi-centre study [13], the quality of life of critically ill patients before ICU admission was frequently good, and only a small proportion of patients had a low quality of life before admission. This is in accordance with our results, showing that, even after an ICU stay, as many as 73% of patients were judged to have good long-term prognosis.

To reduce the likelihood of a poor outcome on the ward, an outreach team has been developed in some hospitals [7], but checking the whole hospital population to detect warning signals can be an overwhelming task for such teams. Our ICU discharge scoring system may help to tailor the profile of patients who would benefit most from outreach team surveillance during their ward stay [7]. This classification system may reduce the burden for the outreach team and allow sustainability despite the global shortage of trained personnel.

## Conclusion

We conclude that, in the setting of our critical care organisation, the vast majority of post-ICU mortality refers to patients with very poor prognosis, while very few patients with good prognosis die in the ward after ICU discharge. The Sabadell scoring system at ICU discharge, a McCabe score modification, is a good stratification tool clearly correlated with hospital outcome.

## Key messages

• Very few patients with good prognosis (< 2%) die in the ward after ICU discharge.

• At ICU discharge, the subjective perception of physicians is a good stratification tool clearly correlated with hospital outcome.

## Abbreviations

APACHE = Acute Physiologic and Chronic Health Evaluation; ICU = intensive care unit.

## Competing interests

The authors declare that they have no competing interests.

## Authors' contributions

RF and FB were responsible for the study concept, data acquisition, data interpretation, and drafting of the manuscript. GN was involved in the data acquisition and in data presentation. AA contributed to data interpretation and writing of the manuscript. All authors read and approved the final manuscript.

## Figures and Tables

**Table 1:** Clinical characteristics of the patients classified into the four expected outcome categories of the Sabadell score at intensive care unit discharge

	Good prognosis (0 points) (*n *= 843)	Long-term poor prognosis (1 point) (*n *= 186)	Short-term poor prognosis (2 points) (*n *= 95)	Expected hospital death (3 points) (*n *= 32)
Age (years)	57 ± 18.3^b,c,d^	67 ± 13.0^a^	70 ± 13.8^a^	72 ± 10.4^a^
Source of admission				
Emergency room	529 (63%)	111 (60%)	56 (59%)	22 (69%)
Surgery	142 (17%)^d^	28 (15%)^d^	14 (15%)^d^	0 (0%)^a,b,c^
Ward	115 (14%)	37 (29%)	22 (23%)	7 (22%)
Other	57 (7%)	10 (5%)	3 (3%)	3 (10%)
Admission diagnosis				
Cardiac	234 (28%)	57 (31%)	23 (24%)	15 (47%)
Respiratory	74 (9%)	45 (24%)	18 (19%)	4 (12%)
Neurological	53 (6%)^d^	10 (5%)^d^	11 (12%)^d^	7 (22%)^a,b,c^
Trauma	142 (17%)^b,c,d^	6 (3%)^a^	5 (5%)^a^	2 (6%)^a^
Other	340 (40%)^d^	68 (36%)^d^	38 (40%)^d^	4 (12%)^a,b,c^
Severe comorbidities (APACHE II score)	160 (19%)^b,c^	93 (50%)^a,d^	45 (47%)^a,d^	8 (25%)^b,c^
'Do-not-resuscitate' orders on admission	0 (0%)	2 (1%)	6 (6%)	3 (10%)^a,b^
APACHE II risk of death (%)	17 ± 18.3^c,d^	25 ± 20.2^d^	32 ± 24.7^a^	39 ± 24.6^a^
Need for vasoactive drugs (*n *(%))	211 (25)^d^	63 (34)^d^	40 (42)^d^	20 (63)^a,b,c^
Tracheal intubation (*n *(%))	303 (36)^c,d^	80 (43)^d^	56 (59)^a^	25 (78)^a,b^
Tracheostomy (*n *(%))	34 (4)^c,d^	17 (9)^c,d^	23 (24)^a,b,d^	15 (47)^a,b,c^
Blood transfusion (*n *(%))	185 (22)	46 (25)	34 (36)	8 (25)
Acute renal failure (*n *(%))	67 (8)^d^	20 (11)^d^	10 (11)^d^	8 (25)^a,b,c^
ICU-acquired infection (*n *(%))	59 (7)^c,d^	19 (10)^c^	26 (27)^a,b^	7 (22)^a^
ICU readmission (*n *(%))	18 (2%)	6 (3%)	4 (4%)	0
Ward mortality (*n *(%))	14 (2%)^b,c,d^	32 (17%)^a,c,d^	39 (41%)^a,b,d^	26 (81%)^a,b,c^

APACHE, Acute Physiologic and Chronic Health Evaluation; ICU, intensive care unit. Comparison of variables by analysis of variance: ^a^*P *< 0.05 compared with good prognosis, ^b^*P *< 0.05 compared with bad prognosis in the long term, ^c^*P *< 0.05 compared with bad prognosis in the short term, ^d^*P *< 0.05 compared with expected hospital death.

**Table 2:** Variables selected by univariate analysis as associated with ward mortality after intensive care unit discharge

	Odds ratio	95% confidence interval	*P *value
At intensive care unit admission			
Chronic renal failure	2.9	1.5–5.8	0.003
Cardiopulmonary resuscitation before admission	3.6	1.7–7.4	0.001
Infection before admission	1.9	1.2–2.9	0.008
Severe coma on admission	3.6	2.1–6.3	0.001
During intensive care unit stay			
APACHE II risk of death (each point)	1.03	1.02–1.04	0.001
Mortality Prediction Model risk of death (each point)	1.03	1.02–1.04	0.001
Sabadell score (each point)	6.1	4.6–8.1	0.001
Intensive care unit acquired infection	2.9	1.7–4.8	0.001
Tracheal intubation	2.3	1.6–3.5	0.001
Reintubation	3.5	1.7–7.1	0.002
Tracheostomy	6.2	3.8–10.3	0.001
Noninvasive ventilation	2.1	1.2–3.3	0.008
Blood transfusion	1.8	1.2–2.8	0.006
Vasoactive drugs	2.5	1.7–3.7	0.001
Parenteral nutrition	2.4	1.3–4.2	0.005

APACHE, Acute Physiologic and Chronic Health Evaluation.

**Table 3:** Multivariate analysis of risk factors associated with ward mortality

Variable	Odds ratio (95% confidence interval)	*P *value
Model before including the Sabadell score		
Age (years)	1.05 (1.03–1.07)	<0.001
APACHE II risk of death	1.01 (1.003–1.025)	<0.01
Tracheostomy	0.20 (0.11–0.37)	<0.001
Model including the Sabadell score as a categorical variable		
Age (years)	1.02 (1.003–1.041)	<0.02
Sabadell score of 1	10.1 (5.2–19.5)	<0.001
Sabadell score of 2	33.2 (16.7–65.9)	<0.001
Sabadell score of 3	202.5 (71.2–575.9)	<0.001

APACHE, Acute Physiologic and Chronic Health Evaluation.
